# How Prediction Errors Shape Perception, Attention, and Motivation

**DOI:** 10.3389/fpsyg.2012.00548

**Published:** 2012-12-11

**Authors:** Hanneke E. M. den Ouden, Peter Kok, Floris P. de Lange

**Affiliations:** ^1^Donders Institute for Brain, Cognition and Behaviour, Radboud University NijmegenNijmegen, Netherlands; ^2^Center for Neural Science, New York UniversityNew York, NY, USA

**Keywords:** prediction, prediction error, expectation, predictive coding, learning, perceptual inference, decision-making

## Abstract

Prediction errors (PE) are a central notion in theoretical models of reinforcement learning, perceptual inference, decision-making and cognition, and prediction error signals have been reported across a wide range of brain regions and experimental paradigms. Here, we will make an attempt to see the forest for the trees and consider the commonalities and differences of reported PE signals in light of recent suggestions that the computation of PE forms a fundamental mode of brain function. We discuss where different types of PE are encoded, how they are generated, and the different functional roles they fulfill. We suggest that while encoding of PE is a common computation across brain regions, the content and function of these error signals can be very different and are determined by the afferent and efferent connections within the neural circuitry in which they arise.

## Introduction

Our ability to perceive structure and predict future states in the world is a remarkable feat that evolution has bestowed upon us. Recent theories have gone as far as surmising that the primary function of the neocortex may lie in the prediction of future states of the environment (Hawkins, 2004; Friston, 2005; Bar, 2009). These theories propose that the brain’s primary objective is to infer the causes of its sensory input by reducing surprise, in order to allow it to successfully predict and interact with the world. In support of these theories, there are many striking examples of the predictive nature of neural computations, in visual (Bar et al., 2006; Alink et al., 2010; Meyer and Olson, 2011), auditory (Ulanovsky et al., 2003; Baldeweg, 2006; Todorovic et al., 2011), and somatosensory perception (Akatsuka et al., 2007; van Ede et al., 2011), as well as in action (Blakemore et al., 1998; Bestmann et al., 2008; Franklin and Wolpert, 2011), language (Kutas and Hillyard, 1980), memory (Erickson and Desimone, 1999; Kumaran and Maguire, 2006, 2009), cognitive control (Alexander and Brown, 2011), and motivational value processing (Schultz, 1998; Hare et al., 2008; Daw et al., 2011). Since the world is a continuously changing and stochastic environment, these predictions likewise need to be continuously changed and fine-tuned on the basis of novel information, which may conflict with the organism’s prior expectations. When such a mismatch between prior expectations and reality arises, this is referred to as a prediction error.

The study of prediction and prediction error signals in the brain is encountered in the largely segregated research fields of motivational control and perception. Prediction errors (PEs) are prominent in models of perception (Rao and Ballard, 1999; Lee and Mumford, 2003), which propose how prior expectations help us to make sense of our environments. In these models, predictions of impending perceptual events help us quickly interpret and disambiguate noisy and ambiguous input (Kersten and Yuille, 2003; Sterzer et al., 2008). Predictions and PEs are also key concepts in models of reward learning, motivational control, and decision-making (e.g., Rescorla and Wagner, 1972; Pearce and Hall, 1980; Behrens et al., 2007; Niv and Schoenbaum, 2008). These models describe how we learn where the bad things lurk and the good things live, and which actions to undertake to avoid them or seek them out respectively (Schultz et al., 1997; Schultz and Dickinson, 2000; Wise, 2004).

In fact, PEs are reported in a bewildering breadth of (hundreds of) studies. While these are all referred to as PEs, the signals reported and discussed in the fields of perception on the one hand and motivational control and learning on the other, do not appear to be of the same nature or serve the same functions. Nevertheless, recent experiments and theorizing suggest that coding of PEs does reflect a general neural coding strategy (Friston, 2005; Clark, 2012). We will build on these ideas and aim to bring closer these disparate literatures on the role of PEs in perception and motivational control (Bromberg-Martin et al., 2010; Redgrave et al., 2011). In the first section, we will give a brief overview of the main “classes” of PEs that have been reported, where we will focus on sensory cortical PEs versus motivationally valenced subcortical PEs. Next, we will look in detail at where and via which neurophysiological mechanisms these classes of PEs are generated. Finally, we will look at what roles PEs play in perception, attention, and motivational control and how they help us to successfully interact with a continuously changing world. We will outline how the same fundamental computational operations can give rise to different functions and highlight the importance of considering the neural circuits in which the PEs arise.

## What are Prediction Errors, and Where in the Brain are they Encoded?

Principally, a prediction error can be defined as the mismatch between a prior expectation and reality. Prior expectations are based on an agent’s model of the world, which is partly hard-wired in the structure of neural circuits and partly derived from statistical regularities in the sensory inputs that the agent experiences over a lifetime. As such, a PE signals a deviation of the current state with respect to what is predicted based on the current model of the world, and calls for an update. Exposure to experimentally manipulated environments indeed alters prior expectations, even when such expectations are the result of a lifetime of experience, such as the prior that light comes from above (Adams et al., 2004), or that slow motion speeds are more likely than fast ones (Weiss et al., 2002; Sotiropoulos et al., 2011). The notion of the coding of PEs as a ubiquitous strategy is supported by the observation that PE signals appear ubiquitously throughout the brain, in relation to the processing of sensory signals, value, motor actions, and cognitive control. One may object that, if PEs are so general, to the degree that all our brains do is code predictions and PEs – then how can we achieve such extensive neural specialization for actions, emotions, and generally functional specialization of areas? The answer lies in the notion that, while coding in terms of PEs is a common currency across brain regions, the exact content and nature of these error signals vastly differs between areas and functional specializations. Below, we will first discuss two main classes of PEs that have been reported in the last decades. Roughly, these can be divided into on the one hand perceptual and cognitive PEs, which report the degree of surprise with respect to a particular outcome, and on the other hand motivational PEs, which also report the valence (sign) of a PE, i.e., not only whether the outcome was surprising, but also whether it was better or worse than expected.

### Perceptual prediction errors

One of the most basic and robust paradigms to demonstrate neuronal responses to unexpected stimuli is the oddball paradigm. Here, presentation of a deviant oddball stimulus in a sequence of repeated standard stimuli elicits larger neural activity over sensory areas. This has become known as the mismatch negativity (MMN) in electrophysiological research, because of a larger deflection of a negative-going evoked potential, when measured with EEG. The MMN was first described in the auditory domain (Näätänen, 1990), and has later also been observed in the visual (Stagg et al., 2004) and somatosensory (Akatsuka et al., 2007) modalities.

**Figure 1:**
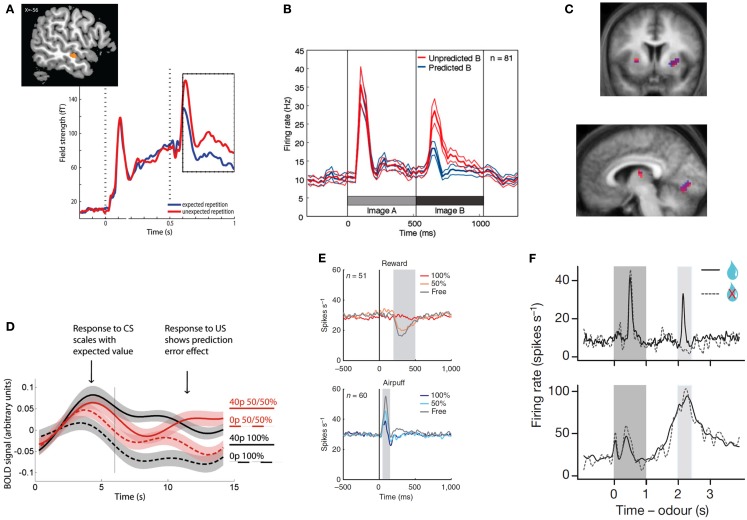
**Top row: examples of unsigned prediction errors**. **(A)** MEG: larger evoked activity in the auditory cortex for repeated but unexpected auditory stimuli. Reprinted from (Todorovic et al., 2011) with permission from the authors. **(B)** Single-unit recordings: larger population firing rate in the inferotemporal cortex for unexpected images. Reprinted from (Meyer and Olson, 2011) with permission from the authors. **(C)** fMRI: correlation between the degree of surprise evoked by a (present or absent) visual stimulus and striatal (top and bottom left) and primary visual (bottom right) hemodynamic activity. Reprinted from (den Ouden et al., 2009) with permission from the authors. **Bottom row: examples of signed prediction errors:** **(D)** fMRI: increased hemodynamic activity in the VTA for outcomes that are better than expected, but decrease for worse than expected. Reprinted from (Klein-Flugge et al., 2011), copyright (2011) with permission from Elsevier. **(E)** Single-unit recordings: neurons in the lateral habenula signal punishment prediction errors, as they fire stronger for outcomes that are worse than expected, and less for outcomes that are better than expected, both in the rewards (top) and punishment (bottom) domain. Reprinted by permission from Macmillan Publishers, Ltd: nature Neuroscience (Matsumoto and Hikosaka, 2009a), copyright (2009). **(F)** Single-unit recordings. Top panel: firing rate of dopaminergic neurons in the VTA at the time of the reward signaling cues (dark gray), and presentation versus omission of reward (light gray block). Bottom panel: GABAergic neurons fire in response to the predictive peaking at the time of the predicted reward, independently of the nature of the outcome (reward or no reward). Reprinted by permission from Macmillan Publishers, Ltd: nature (Cohen et al., 2012), copyright (2012).

The MMN was originally interpreted as resulting from change detection, when a physically different stimulus followed a series of physically identical stimuli. However, subsequent evidence has shown that the MMN is different from simple repetition suppression, and is the result of a violated prediction, rather than a physical stimulus change (Summerfield and Koechlin, 2008; Todorovic et al., 2011; Bendixen et al., 2012; Wacongne et al., 2012; Figure 1A). For example, when a series of rising tones is expected, an MMN is observed when two identical tones are played in succession (Tervaniemi et al., 1994). Also, the neural effects of expectation and repetition appear distinct: when orthogonally manipulating stimulus expectation and stimulus repetition, neural responses in a very early time window (40–60 ms) are attenuated by stimulus repetition but not stimulus expectation, while neural responses in a later time window (100–200 ms) are modulated by expectation but not repetition (Todorovic and de Lange, 2012).

Perceptual PE responses can also be dissociated from related concepts like adaptation and stimulus-driven attention in so-called omission paradigms, in which the neural response to a predicted but withheld event is measured. Remarkably, there is a robust cortical response to such surprising stimulus omissions in the relevant sensory cortical areas (den Ouden et al., 2009; Todorovic et al., 2011; Wacongne et al., 2011; Kok et al., 2012b). Unlike the reduced response for repeated items, it is difficult to explain these brain responses to surprising omissions in terms of stimulus adaptation, since there is no physical stimulus presented. Similarly, while larger neural responses to surprising stimuli can potentially be explained by larger bottom-up (stimulus-driven) attention, this account fails to explain the larger activity for surprising omissions, given the absence of a stimulus that one could attend to.

Although examples of perceptual surprise responses such as the MMN are abundant in primary sensory cortices (den Ouden et al., 2009; Alink et al., 2010; Kok et al., 2012a; Figure 1C), they are also widely reported in more specialized visually responsive regions. For example, Meyer and Olson observed a transitional surprise effect in inferotemporal cortex, the terminus of the ventral stream, where object-selective neurons exhibited a much stronger response to unpredicted than predicted images after image transitions were learned by associative pairing (Meyer and Olson, 2011, Figure 1B).

Finally, PE responses have been reported not only within, but also between sensory modalities, most notably between the auditory and visual domain. For example, in audiovisual speech perception, visual input (lip movements) predicts auditory input (speech sounds), and artificial incongruence between the two has been shown to distort speech perception (McGurk and MacDonald, 1976) as well as increase neural activity in the superior temporal sulcus, a well-known multisensory region (Arnal et al., 2009, 2011). In line with predictive coding accounts, the more predictive a visual stimulus is of the subsequent spoken syllable, the stronger the response in superior temporal sulcus when this prediction is violated (Arnal et al., 2009). Interestingly, this PE response is accompanied by increased functional connectivity between superior temporal sulcus and (unimodal) auditory and visual sensory regions, as well as increased gamma-activity in these lower order sensory regions (Arnal et al., 2011). These results are suggestive of predictive hierarchical message-passing across modalities: the increased gamma-activity reflects increased ascending information from the unimodal sensory regions (Arnal and Giraud, 2012) which send a PE to the superior temporal sulcus.

One may wonder why these PE or surprise responses should be present in so many neural structures along the ventral visual stream, from primary sensory cortices to inferotemporal cortex and hippocampus. In other words: Why do we need so much “PE” signaling? It is important to realize here that these PEs are not simply an unspecific “surprise” response, but that they are linked to a particular representation or prediction. As such, a PE response in a V1 neuron signals surprise about the unexpected presence (or absence) of an oriented edge in a particular part of the visual field, whereas surprise responses in inferotemporal neurons pertain to surprise with respect to highly specific categories of objects. In line with this, Peelen and Kastner (2011) showed that the activity pattern of omissions contains information about the identity of the absent stimulus. Therefore, perceptual PEs do not merely signal surprise, but have representational content.

### Cognitive prediction errors

Cortical areas that are not considered to be part of a sensory processing stream, but instead operate on higher-order representations have also been shown to be sensitive to both predictability and surprise. For example the anterior hippocampus shows larger activity for statistically structured compared to random sequences (Turk-Browne et al., 2009), and exhibits largest activity for unexpected sequences of events, i.e., when predictions about how future events will unfold are violated (Kumaran and Maguire, 2006, 2009). A recent study also suggests that the hippocampus stores and generates predictions of complex visual shapes, in line with the larger activity for unexpected shapes observed by earlier studies (Schapiro et al., 2012). This study found that the exact activity *patterns* in the hippocampus to different stimuli became more similar after a learning phase in which these stimuli were paired. Interestingly, this shaping was “forward-looking/predictive” in the CA2/3 and dentate gyrus sub-regions of the hippocampus. In this experiment, two stimuli (A and B) were paired together, such that A was likely to be followed by B (A → B), but B was unlikely to be followed by A. After learning, the activity pattern for stimulus A (A-post) became more similar to the activity pattern for stimulus B before learning (B-pre). However, the activity pattern for B after training (B-post) did not become more similar to the activity pattern to A before learning (A-pre), which excludes an explanation in terms of simple association. Even higher in the cortical hierarchy and further away from the “sensory” PEs are the “cognitive” PE signals, repeatedly observed in the dorsolateral prefrontal cortex. In various paradigms, in which subjects need to predict outcome stimuli based on previously learned associations, the dorsolateral prefrontal cortex strongly responds to abstract rule violations, independently of stimulus novelty (Fletcher et al., 2001; Corlett et al., 2004; Turner et al., 2004).

### Motivational prediction errors

The sensory and higher-order cognitive PEs described above reflect only one aspect of the mismatch between a prediction and the outcome, namely the size of this mismatch. In perceptual inference, an outcome can be more or less surprising, but never better or worse than expected. These PEs, which do not reflect the valence of the outcome but simply the surprise engendered by this outcome, are often referred to as “unsigned” PEs. However, in order to learn and use PEs to guide motivational action, not only the size but also its valence (i.e., sign) of the PE is of relevance. In other words, the PE has to reflect also whether an outcome was better or worse than expected.

Signed PEs play a central role in many computational models of reinforcement learning. These models describe how an agent learns the value of actions and stimuli in a complex environment, and signed PEs that contain information about the direction in which the prediction was wrong, serve as a teaching signal that allows for updating of the value of the current action or stimulus. In a seminal series of studies, Schultz and colleagues showed that the firing pattern of phasic dopamine neurons in the macaque ventral tegmental area (VTA) reflects exactly such reward PEs (Romo and Schultz, 1990; Mirenowicz and Schultz, 1994, 1996; Schultz, 1998). These neurons (i) increase their firing rate when an outcome was better than expected, (ii) do not change when the outcome was expected, and (iii) fall silent when an expected reward is omitted (Figures 1D–F). This neuronal behavior supports the hypothesis that the dopamine neurons in the VTA signal reward PE (Schultz and Dickinson, 2000). Computational reinforcement learning models propose that PEs in part determine the size and direction of the update of the prediction engendered by the cue (Rescorla and Wagner, 1972; Schultz and Dickinson, 2000), and as such play a central role in motivational learning.

Inspired by the results from these animal experiments, fMRI studies have subsequently shown that also in humans the VTA responds to the difference between expected and actual rewards (D’Ardenne et al., 2008; Klein-Flugge et al., 2011). Additionally, many human fMRI studies reported reward PE responses in the ventral striatum for a wide range of stimuli including attractive faces, money, and food (e.g., Pagnoni et al., 2002; McClure et al., 2003; O’Doherty et al., 2003; Abler et al., 2006; Rodriguez et al., 2006; Bray and O’Doherty, 2007; Seymour et al., 2007; Yacubian et al., 2007; Hare et al., 2008; Niv et al., 2012).

Complementing research on reward PEs, there is some evidence for neurons or neuronal populations responding in a manner consistent with punishment PEs. Neurons in the primate lateral habenula, for example, show increased activity after an unexpected punishment, and decreased activity after an unexpected reward (Matsumoto and Hikosaka, 2007, Figure 1E). Such punishment PEs are also observed in the VTA, possibly resulting from projections from the lateral habenula to VTA GABAergic neurons (Cohen et al., 2012). Using fMRI, punishment PEs have also been reported in the human striatum (Seymour et al., 2007) and in the amygdala (Yacubian et al., 2006).

The unsigned PEs described in the previous sections on perceptual and cognitive PEs were all observed in cortical regions, whereas the signed reward and punishment PEs in this section were generally reported from subcortical areas. This may create the impression of a cortical/subcortical divide with respect to the information content of the prediction errors. However, subcortical unsigned PEs to valenced stimuli have also been reported, notably in the primate midbrain dopamine neurons in the substantia nigra. These neurons increase their firing rate to unexpected stimuli, independent of the valence (reward or punishment; Matsumoto and Hikosaka, 2009b). Similarly, a hemodynamic correlate of unsigned PEs has also been observed in the human brain even in response to affectively neutral stimuli, both in the striatum (Zink et al., 2003; den Ouden et al., 2009, 2010), and in the VTA (Bunzeck and Düzel, 2006). Although rarer, signed motivational error signals have also been reported in cortical areas, including in the orbitofrontal cortex to reward (Takahashi et al., 2009; Sul et al., 2010), the insular cortex to punishment (Pessiglione et al., 2006), and in the medial prefrontal cortex to both positive and negative feedback (Matsumoto et al., 2007).

## How are Prediction Errors Generated?

As discussed above, PEs appear ubiquitously throughout the brain, lending support to the notion that coding of PEs is a general neural coding strategy (Friston, 2005; Clark, 2012). In models of predictive coding, the brain constructs a generative model of how causes in the world elicit sensory inputs. Then, given some sensory inputs, this model can be inverted to recognize the causes of these inputs. In this scheme, each level of the processing hierarchy receives bottom-up sensory input from the level below and top-down predictions from the level above. Prediction error, i.e., the difference between the true and estimated probability distribution of the causes, is minimized at all levels of the hierarchy by adjusting connection strengths through synaptic plasticity (Friston, 2005). In this next section we will discuss in more detail how these PEs may be generated.

### Cortical prediction errors: Layers and columns

**Figure 2:**
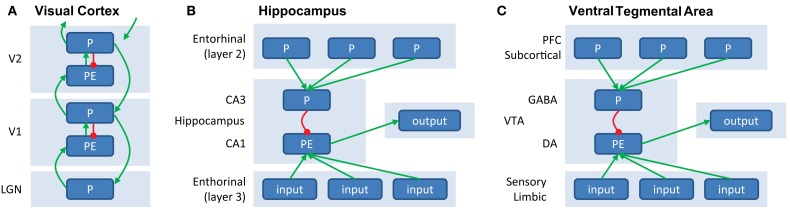
**(A)** Generation of prediction errors within a cortical ensemble. PEs are generated by mismatch between predictions (P, in agranular layers, inhibitory) and input (originating from L2/3 from lower unit, arriving in L4, excitatory). The PE unit therefore reflects the difference between input and prediction, and activity in P units will be updated to minimize this discrepancy. Predictions (P) are both sent forward as input to a hierarchically higher level (via supragranular layers, L2/3) and backward to update predictions at a lower level (via infragranular layers, L5/6). **(B)** Generation of PEs within the hippocampus. Predictions, based on stored memories drive CA3 via layer 2 of the entorinal cortex. CA3 provides an inhibitory signal to CA1. At the same time, sensory inputs from layer 3 of the entorhinal cortex provide excitatory input to CA1, which is thought to serve as a “comparator” between predictions and input. The resulting mismatch is sent as output to (a.o.) VTA. **(C)** Generation of PEs within VTA. VTA GABAergic neurons exert an inhibitory influence that counteracts the driving excitatory input from primary rewards when the reward is expected, see also Figure 1F.

Contemporary predictive coding models (Rao and Ballard, 1999; Friston, 2005; Spratling, 2008) posit the existence of separate “representation” or “prediction” units (P) and PE units within each cortical column. In these models, the cortical column is considered the basic computational module (Mountcastle, 1997; Bastos et al., 2012, although see Horton and Adams, 2005). There are intrinsic (within the cortical column) and extrinsic (between columns) connections between P and PE units (Figure 2A). There are several flavors of predictive coding architectures, and while these models differ in terms of their exact connectivity pattern, at the heart of all of these models are the interactions between excitatory and inhibitory P and PE units (Spratling, 2008).

Figure 2A illustrates a potentially neurophysiologically plausible implementation of message-passing within and between P and PE units. Here, PEs could be generated in the granular layer (L4) of a cortical column, by subtracting the prediction (P) response from the agranular layers from the input (which is provided by the lower level). Large PEs lead to updating of predictions, which are then sent forward as input to higher cortical areas (via superficial layers, L2/3), as well as backward to update predictions in lower areas (via deep layers, L5/6). In this scheme, the excitatory feedback from higher to lower P units can be thought of as activating hypotheses, and provides a natural explanation for phenomena such as “omission responses” (see paragraph on “Perceptual PEs” earlier). Namely, predictions (P units) at higher levels activate predictions (P units) at lower levels, giving rise to a neural response even when no stimulus is presented but a stimulus is expected. While this scheme differs in some respects to implementations suggested previously (Rao and Ballard, 1999; Friston, 2005), it has been shown to be mathematically equivalent under some simplifying assumptions (Spratling, 2008) and relies on the same general principle of inhibition of PE units by P units. Note that this message-passing scheme is not necessarily restricted to early sensory cortex (where it has been studied in most detail), but is thought to represent a general coding scheme of cortico-cortical interactions.

Although the scheme described above is appealing, future empirical work on the timing of activity in different layers, as well as a fuller understanding of the intrinsic and extrinsic connectivity patterns within and between cortical units is sorely needed to provide a stronger empirical basis for this theoretical proposition. More generally speaking, since there is at present no direct evidence for distinct P and PE units within the cortical hierarchy (Summerfield and Egner, 2009), it will be imperative for future studies to concurrently measure several cortical units with greater (laminar) specificity, in order to further elucidate the actual neural implementation of the communication between potential P and PE signals.

### Subcortical prediction errors

A similar integration of feedback predictions and feedforward inputs is thought to give rise to the generation of PEs in subcortical circuits. An elegant recent optogenetic study showed in detail a mechanism at the level of the microcircuits that underlies the generation of a dopaminergic reward PE signal in the VTA (Cohen et al., 2012). This study revealed that a top-down inhibitory input on the dopaminergic neurons in the VTA in proportion to the expected reward is present during the delay between a predictive cue and the outcome, as previously proposed by theoretical models (Schultz et al., 1997). While dopaminergic VTA neurons showed responses consistent with coding of reward PEs, GABAergic neurons showed persistent activity during the delay between a reward-predicting cue and the outcome. This activity was not sensitive to the actual outcome, i.e., whether the reward was delivered or omitted, but was proportional to the reward prediction engendered by the cue. Thus, VTA GABAergic neurons provide an inhibitory input counteracting the bottom-up “drive” from expected but not unexpected rewards. These GABAergic VTA neurons in turn receive prefrontal and subcortical inputs, which could relay the reward prediction signals engendered by the cues (Matsumoto and Hikosaka, 2007; Takahashi et al., 2011). Thus, top-down cortical and subcortical predictions may set a threshold via GABAergic VTA neurons that the bottom-up primary reward signals that are driving the dopaminergic VTA neurons need to overcome. Negative reward PEs observed in the VTA neurons, i.e., a dip in response to a punishing event, could be driven by lateral habenula neurons that respond to aversive events and project to the VTA GABAergic neurons (Jhou et al., 2009).

In the previous example, the PE was computed within the midbrain itself. However, subcortical PE signals may also arise as input signals from cortical areas. For example, the hippocampus induces novelty-dependent dopaminergic responses in the VTA (Lisman and Grace, 2005). Here, area CA1 of the hippocampus acts as a “comparator,” of predictions from neurons in CA3, triggered by sensory cues, to “reality” from sensory cortical inputs. A second example are the unsigned PEs at very short latencies in the dopaminergic neurons of the substantia nigra pars compacta (Matsumoto and Hikosaka, 2009b) that are a result of projections from the primary sensory superior colliculus, which signals novel, salient, and unexpected events in a retinotopic fashion (Comoli et al., 2003).

### A fundamental computation

Having taken a closer look at the mechanisms by which PEs are generated in both cortical and subcortical structures, it appears that there are some universal mechanisms underlying the generation of PEs. In short, feedback “prediction units” (e.g., deep layers in hierarchically higher visual areas, CA3, prefrontal cortex) set an activity pattern that is integrated with feedforward inputs in “PE units” (e.g., granular layer in hierarchically lower visual areas, CA1, VTA), to reflect the difference between prediction and reality (Figure 2). The exact content and nature of the PEs is determined by the neural circuitry in which the PEs arise. Within the visual processing hierarchy, predictions concern primary visual “currencies” such as orientation and contrast, and therefore PEs will reflect surprise about orientation and contrast. These PEs percolate up to and are integrated in messages sent to higher-order areas, where predictions pertain to, for example, faces and houses, and PEs will reflect surprise about faces and houses. In limbic areas, feedback predictions will concern expectations about good and bad upcoming events, and so PEs will reflect surprise in this domain. Similarly, the role that a particular PE plays will depend crucially on the neural circuit in which it arises: PE signals that are projected to sensory areas are in a position to affect perceptual inference, whereas PE signals arriving in motor areas can (more directly) affect behavior. In the next section we will discuss various neural circuits and the different functions that PEs may fulfill.

## How are Prediction Errors Used?

The exact role a PE plays depends on several factors. First, the information content carried by PEs affects how they may be used. Unsigned perceptual PEs carry information about the surprising presence or absence of a stimulus feature in the visual scene (i.e., represent the difference between current predictions and sensory input), and thereby allow to update the current model of the world. Signed reward PEs contain information about the direction of the error, and therefore can afford learning by signaling the direction of an update, or motivational processing by signaling the valence of an outcome. Second, PE neurons can affect post-synaptic signaling on different timescales. Post-synaptic effects may be short-lived and directly affect perception, behavior or attention, or they might control storage and updating of predictions by inducing changes in synaptic strength.

In this section, we will discuss the various roles cortical and subcortical PEs may play. We discuss three main functions of PEs. First we will discuss how perceptual PEs aid us to rapidly make sense of sensory inputs, i.e., perceptual inference, and how PEs are crucial in shaping of internal generative models of the world that allow us to interact with the world. Next we will discuss how these sensory and higher-order cortical PEs can alert us to unexpected events and allow for reorienting responses. Then we will zoom in on the role of PEs in motivational learning and action selection. We will use the role of PE signals in the basal ganglia in selection of cortical representations to illustrate how the same fundamental computation may lead to different functional results, underlining the importance of taking into account the neural network in which a PE arises. Finally, we will discuss how the impact of PEs may depend on not only on their magnitude, but also on their precision.

### Perceptual inference

The role of PEs in inference has been elaborated on most extensively in the domain of perception. Helmholtz famously viewed perception as the generation of a best guess (i.e., inference) about the state of the world, in view of the data (Von Helmholtz, 1867). In other words, the brain creates an internal generative model of the world that embodies a prediction of what will be observed next. PEs can then be seen as a measure of how good such a guess is; iterative hypothesis testing (i.e., PE minimization) across the cortical hierarchy will result in the best possible explanation of the input given the agent’s generative model. Crucially then, the brain’s generative models shape perception; what we perceive is that part of our model of the world that best fits current inputs and expectations, rather than simply an accumulation of sensory evidence. This view is corroborated by the fact that in the brain, “waves” of feedforward and feedback activity have been observed (Lamme and Roelfsema, 2000) and that feedback connections greatly outweigh feedforward connections, even in very basic sensory areas such as between V1 and LGN (Peters et al., 1994). Functionally, it has been shown that processing in even the earliest stages of the cortical hierarchy is affected by prior expectations (den Ouden et al., 2009; Alink et al., 2010; Todorovic et al., 2011; Kok et al., 2012a). Specifically, valid prior expectations allow for selection of the proper hypotheses (i.e., activating relevant P units) in advance of stimulation, facilitation of perception (Bar, 2004), and enhancement of neural representations of stimuli, while reducing the amount of processing required. Empirical support is provided by a recent behavioral/modeling study which has found that the effects of prior expectations on contrast sensitivity are well explained by an increase of baseline activity in signal-selective sensory neurons, reflecting the activation of P units (Wyart et al., 2012). This issue was also tackled by a recent fMRI study in our lab that manipulated subjects’ expectations about upcoming visual stimuli, and probed the effects this had on neural activity in the primary visual cortex (V1; Kok et al., 2012a). Crucially, this study investigated not only the amplitude of the neural response evoked by stimuli, but also used multivoxel pattern analysis to study the effect of expectation on the amount of information contained in the neural signal. Interestingly, a valid expectation led to a *decrease* in the amplitude of the neural signal evoked by stimuli in V1, but an *increase* in the amount of stimulus information contained in the signal. That is, when the proper hypothesis is selected prior to stimulation, less message-passing across the hierarchy is needed to settle on the best explanation of the incoming data, allowing the brain to settle on the best explanation of the incoming data more efficiently. Such neural representations are also more unequivocal, since hypotheses that are not pre-selected are not tested, unless there is strong evidence for them in the input, i.e., unless the prior expectation was invalid and large PEs ensue. Therefore, when sensory information is ambiguous, prior information can serve to disambiguate perception (Sterzer et al., 2008; Hsieh et al., 2010; Wyart et al., 2012).

Further empirical evidence for the shaping of internal generative models based on the statistics of the world comes from a study that used neural recordings in ferrets (Berkes et al., 2011). These authors investigated spontaneous cortical activity in early visual cortex, in absence of any visual stimulation, and compared this to visual activity evoked by natural scenes. Interestingly, the similarity between these activity patterns increased with age, and was specific to responses evoked by natural scenes. These results suggest that the spontaneous fluctuations in the visual cortex may embody internal models, which are optimally adapted to the statistics of the environment as the animals learn to navigate their environment.

### Alerting and orienting

Perceptual PEs have also been proposed to signal salience (Spratling, 2012). Under this account, salience is determined by how unexpected an input is, and not solely by bottom-up stimulus characteristics such as contrast (Li, 2002). In fact, salience arises quite naturally from predictive coding theories of neural processing, since the amplitude of the response a stimulus evokes is directly determined by how unexpected it is. This allows the brain to devote relatively little (attentional and metabolic) resources to expected inputs, such as when driving a car in a familiar environment, without losing sensitivity to potentially vital unexpected inputs such as a deer crossing the road. In fact, silencing expected inputs would make such an unexpected event stand out even more. This benefit is due to the fact that, in predictive coding, silencing occurs on the basis of (learned) expectations, as opposed to attentional suppression of particular parts of the sensory inputs, such as certain spatial locations or features, which would reduce sensitivity for stimuli sharing (some of) those features.

Detection of a salient stimulus can then be used as an alerting/reorienting signal, and relayed to the appropriate nodes that can implement a shift in attention or behavior. In line with this, midbrain dopamine neurons (substantia nigra pars compacta) show a fast (<100 ms) phasic increase in firing in response to salient unexpected sensory stimuli, via a direct projection from the superior colliculus (Comoli et al., 2003; Dommett et al., 2005). These salience-encoding neurons fit well with theories suggesting that dopamine plays an important role in alerting, orienting, and arousing responses (Redgrave et al., 1999; Kapur, 2003; Redgrave and Gurney, 2006). The lateral substantia nigra pars compacta also receives excitatory cortical inputs from somatosensory and motor cortex (Watabe-Uchida et al., 2012), as well as the subthalamic nucleus, which in general responds to sudden changes in the environment as well as various motor and reward events (Matsumura et al., 1992).

The basal ganglia form an important output target of the substantia nigra dopamine neurons, and are in an excellent position to implement a reorienting action in response to a salient stimulus, to enable further processing of this stimulus (Redgrave et al., 2011). We and others have shown hemodynamic activity in response to unsigned PEs in the main input node of the basal ganglia, the striatum (Zink et al., 2003; Wittmann et al., 2007), even to surprisingly absent stimuli (den Ouden et al., 2010). Such fast, unsigned PEs from sensory areas may allow the basal ganglia to act as a circuit breaker, by inhibiting ongoing behavior or processing and allowing for reorienting toward an unexpected stimulus (Redgrave et al., 1999; Bromberg-Martin et al., 2010). In line with these suggestions, we have shown using fMRI that striatal PE activity gates effective connectivity from visual areas to the premotor cortex, upregulating visual input in response to unexpected stimuli, which required an unexpected response (den Ouden et al., 2010). Underlining the universality of the basal ganglia gating function, we also showed that striatal activity to salient stimuli gated prefrontal inputs to visual areas to upregulate processing of the visual stimuli accompanying an attentional shift (van Schouwenburg et al., 2010).

### Motivational control and learning

As mentioned above, reinforcement learning models suggest a crucial role for PEs in learning (Rescorla and Wagner, 1972; Pearce and Hall, 1980). Experimentally, it has been shown that surprise is crucial for learning through a phenomenon called “blocking” (Kamin, 1969). Here, when the presence of a reinforcer can be fully predicted by the cues present, then an additional cue will not become associated with the reinforcer, even if they are paired repeatedly.

Dopaminergic (signed) reward PEs in the VTA have long been suggested to play a crucial role in reinforcement learning (Schultz et al., 1997). Systemic manipulations of dopamine levels in patients and healthy subjects show opposite effects on reward- and punishment-based learning (Frank et al., 2004; Moustafa et al., 2008; Cools et al., 2009; Palminteri et al., 2009). These opposite effects are suggested to reflect modulation of distinct striatal pathways that mediate activation and inhibition of responses via frontostriatal coupling changes (Frank, 2005). A large positive reward PE will strengthen the associated action, whereas a negative reward PE would inhibit actions. This will then result in a selection bias toward the positively reinforced actions in the future. Thus, reward PEs in the basal ganglia lead to both direct motivational effects in terms of action selection, but also to long term learning as a result of a selection bias of reinforced actions.

The role of the striatum as a gateway in the translation of environmental cues into behavioral activation (or inhibition) is not new (Mogenson and Yang, 1991). More recently, it has been proposed that this selection function of the basal ganglia is not limited to action selection or reinforcement learning. Given that the basal ganglia receive cortical, limbic, and brainstem inputs and thus have access to motivational, affective, cognitive, and motor information, they may form the final common pathway where information from a wide variety of sources is integrated and then guide selection among cortical representations, actions and goals. In this view the same computations are performed across the basal ganglia in a consistent but parallel fashion. The critical functional distinction between phenomenologically different functions of the basal ganglia system then stems from the differences in input sources and output targets, not from the fundamental differences in the nature of the performed computational operations themselves.

This fundamental function is the integration and implementation of information to allow PEs to guide switching between cortical representations, actions and goals. This view is supported by the observation that the microcircuitry of the basal ganglia is remarkably preserved across different parts of the striatum, suggesting that the same basic computations are performed, if indeed structure follows function (Pennartz et al., 2011). Functional differences then arise from differences in the input sources and output targets. For example, outcome predictions in dorsolateral striatum will be derived from inputs from sensorimotor areas and will lead to action selection, whereas nucleus accumbens, with inputs from the amygdala and VTA, will report reward PEs in a reinforcement learning task and allow motivational aspects to guide behavior. Hippocampal and prefrontal glutamatergic inputs to the ventral striatum may provide the top-down context information or predictions to modulate neural activity. We provided empirical support for this hypothesized general gating role of the surprise signals in the basal ganglia across both reward and non-reward contexts (den Ouden et al., 2010; van Schouwenburg et al., 2010, in revision). In the first of these studies we showed that PE signals in response to stimuli that required an update were present in the putamen, which is known to connect to cortical motor planning areas (den Ouden et al., 2010). This putamen activity then upregulated the influence of sensory areas to the premotor cortex whenever a surprising motor response was required based on surprising visual input. In two further studies, we showed that more ventral parts of the basal ganglia, which are part of the attentional network, upregulated feedback inputs from prefrontal attentional areas to the visual cortex, in response to attentional shifts, which enhanced processing of newly attended visual stimuli (van Schouwenburg et al., 2010; in revision).

How may these experience-dependent changes in the interactions between prediction and PE units be implemented at a physiological level? Already in 1949, Donald Hebb suggested that changes in connectivity are central to the physiological implementation of association learning, where co-activation of pre and post-synaptic neurons would lead to synaptic strengthening (Hebb, 1949), a principle also known as “firing together results in wiring together.” Predictive coding theories propose that PEs are minimized by adjusting the synaptic efficacies (or connections strengths) both between and within different levels of the processing hierarchy (Figure 2).The glutamatergic NMDA receptor found at excitatory synapses has unique molecular properties that allow it to function as a coincidence detector of afferent and efferent activity, and as such initiate synaptic plasticity (Genoux and Montgomery, 2007). Activation of the NMDA-receptors results in recruitment of a different type of glutamatergic receptors, called AMPA receptors. These post-synaptic AMPA receptors determine the current strength of the excitatory connections (Malinow and Malenka, 2002). Thus activation of the NMDA-receptors by concurrent pre and post-synaptic activity leads to more permanent strengthening of this synapse. Indeed, NMDA-dependent mechanisms have been found to play a key role in plasticity in learning and memory processes in the brain (e.g., see Morris, 1989; Gu, 2002; Ji et al., 2005; Genoux and Montgomery, 2007; Tye et al., 2008). Modulatory neurotransmitters play an important role in strengthening or weakening these effects. For example, dopaminergic firing signaling reward PEs have been proposed to serve as a supervised learning signal determining the weight of the associative strength between actions and stimuli (Schultz and Dickinson, 2000; Friston et al., 2012), as we will discuss in the next section.

### Precision of prediction errors

Since PEs cause us to update our model of the world, either on the short term (inference) or long term (learning), it is important to know how reliable these errors are. For example, it is important to know whether certain sensory inputs fail to match our prior expectations because they contain information that disproves our current hypothesis (e.g., we hear a dog but see a cat), or because the sensory inputs are simply too noisy (we hear a dog but see only mist). While the former should cause us to update our beliefs (a barking cat!), the latter should not. In traditional reinforcement learning models, this dilemma of how to weigh PEs with respect to prior beliefs is solved by the inclusion of a learning rate that determines this relative weight (Rescorla and Wagner, 1972). The importance of the reliability, or precision, of PEs has also been recognized in recent formulations of predictive coding theories (Friston, 2005). Specifically, it has been suggested that PEs are weighted by their precision (i.e., reliability), leading to less weight being put on less reliable sensory information. This means that the brain needs to estimate not only the errors themselves, but also the precision of these errors, and it has been suggested that attention is the process whereby the brain optimizes precision estimates (Friston, 2009; Feldman and Friston, 2010; Hohwy, 2012). By enhancing the precision of specific PEs, attention increases the weight that is put on these errors in subsequent inference and learning. This is equivalent to proposals of attention increasing synaptic gain (precision) of specific sensory neurons (PE units). Note that in predictive coding models, sensory data and PE are equivalent, since these errors are the only sensory information that is yet to be explained.

Empirical support for such a role for attention comes from a recent study showing that while valid prior expectations indeed lead to reduced activity in primary visual cortex, attention can reverse this effect and boost activity in the same region (Kok et al., 2012b). This effect of attention was specific to areas of visual cortex where a sensory input was expected, demonstrating that attention does not indiscriminately increase activity, but does so in conjunction with current expectations. Such a role for attention within a predictive coding framework resolves a seeming contradiction in the literature regarding the effects of expectation on neural activity. Specifically, expectation has often been reported to reduce neural activity when stimuli are task-irrelevant (unattended), but to enhance neural activity when stimuli are task-relevant (attended; Rauss et al., 2011). Such findings sit comfortably within a framework wherein attention boosts PEs related to current expectations.

It has recently been suggested that tonic dopamine firing controls the precision of cues that engender actions (Friston et al., 2012). This account extends the models of predictive coding such that dopamine determines the relative “weight” of feedforward sensory PEs with respect to the predictions. In the original model, superficial pyramidal cells integrate the effects of excitatory inputs encoding feedforward sensory PEs, and inhibitory inputs encoding predictions. Tonic dopaminergic firing in the substantia nigra and VTA then encode the precision of the incoming information and modulate the postsynaptic (dendritic) responses to these PEs in proportion to their precision. This modulation is implemented via widespread dopaminergic projections from the substantia nigra and VTA to the superior colliculus, striatum, and throughout the cortex. In line with this account, dopamine neurons have been reported in the primate midbrain that report uncertainty about the predictions (Fiorillo et al., 2003).

The importance of appropriate precision weighting of PEs becomes clear when one considers what happens when things go awry. For example, one of the mechanisms suggested to be involved in psychosis is aberrant PE signaling (Corlett et al., 2010). During early stages of psychosis, patients often report increased intensity of their perceptual experiences (i.e., brighter colors, louder sounds), consistent with an abnormally large PE (and subsequent increased salience) being evoked by these events, presumably due to an inability to “explain away” sensory inputs through top-down predictions. Indeed, a model for these early stages of psychosis that is often used, is the administration of ketamine, a drug known to block NMDA-receptors (surmised to be involved in top-down predictions), and enhance AMPA receptors (involved in feedforward signaling, and therefore a likely candidate for PE signaling). Such augmented PEs in turn lead to inappropriate updating of these patients’ model of the world, causing delusions to arise in later stages of the disease.

## Conclusion

In this review we aimed to expose the generality and universality of the neural coding of prediction errors. PEs appear to be omnipresent in the brain, as empirical support has been provided in perceptual, attentional, cognitive, and motivational processes in both cortical and subcortical regions. We suggest that the computation of these PEs follows a general principle, where a comparison is made between bottom-up inputs and top-down predictions, and of which the exact form depends on the type of computation (signed or unsigned PEs), and neural structures (cortical or subcortical units). Importantly, PEs are not necessarily a non-specific surprise/arousal signal, but can carry detailed content with respect to *how* the input is surprising, owing to the specific location and connectivity of the PE unit in the cortical hierarchy. Thereby, PEs can update perception, trigger attentional orienting and affect motivational learning and control, as well as initiate the formation of new memories. Finally, the precision (i.e., fidelity, inverse variance) of PEs should be taken into account in deciding how much internal models should be updated. It is speculated that there may be a role for tonic dopamine in controlling this parameter. In summary, while the encoding of PEs is a common currency across brain regions, the exact content and nature of these error signals differs between areas and functional specializations and is determined by the afferent and efferent connections within the neural circuitry in which they arise.

## Conflict of Interest Statement

The authors declare that the research was conducted in the absence of any commercial or financial relationships that could be construed as a potential conflict of interest.
